# Analysis of Unique Motility of the Unicellular Green Alga *Chlamydomonas reinhardtii* at Low Temperatures down to −8 °C

**DOI:** 10.3390/mi15030410

**Published:** 2024-03-19

**Authors:** Kyohei Yamashita, Tomoka Yamaguchi, Shigehiro Ikeno, Asuka Koyama, Tetsuo Aono, Ayaka Mori, Shoto Serizawa, Yuji Ishikawa, Eiji Tokunaga

**Affiliations:** Department of Physics, Faculty of Science Division I, Tokyo University of Science, Shinjuku-ku, Tokyo 162-8601, Japaneiji@rs.tus.ac.jp (E.T.)

**Keywords:** low temperature, motility, *Chlamydomonas reinhardtii*, supercooling, fast Fourier transform (FFT) analysis, viscosity, Ficoll 400, microscale, ImageJ, flagella

## Abstract

Previous studies of motility at low temperatures in *Chlamydomonas reinhardtii* have been conducted at temperatures of up to 15 °C. In this study, we report that *C. reinhardtii* exhibits unique motility at a lower temperature range (−8.7 to 1.7 °C). Cell motility was recorded using four low-cost, easy-to-operate observation systems. Fast Fourier transform (FFT) analysis at room temperature (20–27 °C) showed that the main peak frequency of oscillations ranged from 44 to 61 Hz, which is consistent with the 60 Hz beat frequency of flagella. At lower temperatures, swimming velocity decreased with decreasing temperature. The results of the FFT analysis showed that the major peak shifted to the 5–18 Hz range, suggesting that the flagellar beat frequency was decreasing. The FFT spectra had distinct major peaks in both temperature ranges, indicating that the oscillations were regular. This was not affected by the wavelength of the observation light source (white, red, green or blue LED) or the environmental spatial scale of the cells. In contrast, cells in a highly viscous (3.5 mPa·s) culture at room temperature showed numerous peaks in the 0–200 Hz frequency band, indicating that the oscillations were irregular. These findings contribute to a better understanding of motility under lower-temperature conditions in *C. reinhardtii*.

## 1. Introduction

*Chlamydomonas reinhardtii* is a photosynthetic unicellular green alga, spherical in shape, and approximately 10 μm in diameter, that propels itself through water using two flagella [1,2,3]. The motility of *C. reinhardtii* in still water has been reported, with an average free-swimming velocity of approximately 100 µm/s [4]. Within the cell, a large chloroplast and several mitochondria are contained, and it can accurately detect the direction of light sources through its eyespot. Upon perceiving light, the balance of flagellar beating changes, allowing the cell to alter its swimming direction towards the light source. The beat frequency of the cell adapted to light is around 60 Hz, whereas in darkness, the frequency is approximately 50 Hz [5]. 

*C. reinhardtii* exhibits complex motility behaviors, including chemotaxis [6,7], gravitaxis [8,9], and gyrotaxis [10], allowing it to navigate towards optimal environments based on nutrient levels, salinity, and light. It also shows specific responses to light changes. Photophobic reactions lead to backward swimming when light intensity suddenly increases [11], whereas phototaxis dictates movement towards or away from light, depending on its wavelength (490–520 nm) [12,13]. Although artificial, the induction of a near-stationary rotation by reducing flagella to a single one through genetic manipulation [14] or mechanical methods [15] has been confirmed.

*C. reinhardtii* is recognized for its industrial application as a nutritious food source [16], comparable to well-known microalgae like *Euglena gracilis* [17,18], *Chlorella*, and *Spirulina*. Notably, it surpasses these in starch and lipid content, earning FDA’s GRAS certification for its safety as a food additive [16]. Furthermore, its potential as a functional food that could improve the gastrointestinal health of mice and humans has been elucidated in studies [19]. Additionally, its unique components, such as sulfated polysaccharides, offer antibiotic [20], antioxidant, and anticancer effects [21]. It has been revealed that subjecting *C. reinhardtii* to cold treatment consistently accumulates starch within the cells [22] and increases the content of alpha-linolenic acid, an essential omega-3 fatty acid for humans [23], drawing attention to research on physiological responses to cold [24]. Efforts are also being made to develop large-scale cultivation techniques for commercialization [25].

In the academic field, *C. reinhardtii* plays a significant role as a model organism for studying phototaxis, photosynthesis, and the genetics and physiology of flagellar motility. Research has also been reported on clean energy production, such as hydrogen production [26,27,28,29], and in statistical studies related to the spatial distribution of cells [30]. Particularly in temperature-related research, it has been utilized as a model plant system for examining many aspects of the heat stress response [31,32], and more recently, it has been used in studies of cold-induced responses [24]. In the latter studies, *C. reinhardtii* has been shown to contain both mechanisms common to other plants and species-specific mechanisms of cold tolerance.

*C. reinhardtii* is a mesophilic organism, with an optimal temperature range considered to be between 20 °C and 32 °C. It is known that when the temperature shifts abruptly from the typical growth range to, for instance, 39 °C, an adaptive response to heat shock is triggered [33,34]. Conversely, *C. reinhardtii*, when subjected to moderate cooling stress, exhibits a decrease in growth rate, chlorosis, and progressive membrane and oxidative damage [35], and low-temperature adaptation is induced at temperatures below 7 °C [36]. Severe cold stress below 3 °C can lead to cell death within a short period [22]. Previous studies of physiological responses to cold have primarily focused on biochemical approaches, such as identifying genes and proteins associated with low-temperature stress [37,38] and changes in metabolic processes and metabolites [39]. 

In contrast, physical approaches such as motility analysis, while extensively studied at ambient temperatures [40,41,42], have, to the best of our knowledge, only been conducted at temperatures as low as 15 °C [43]. This is thought to be due to the substantial stress placed on the cells at lower temperatures, making long-term physiological studies challenging. According to previous research, when *C. reinhardtii* was cultured at 4 °C for seven days, the survival rate remained above 90%, but after approximately 80 days of culture, the survival rate dropped to about 3%. Furthermore, when cultured at −2 °C, the survival rate decreased to about 50% after two days and fell to less than 1% after seven days [44]. 

This study focused on the analysis of the motility of *C. reinhardtii* under harsh growth conditions at temperatures around 0 °C or below over a short period. Hereafter, the temperature range of −8.7 to 1.7 °C, or lower, utilized in this research will be defined as “low temperature.” Initially, four types of observation systems (OS) were devised to stably observe cells in a low-temperature environment. These systems are characterized as being low in cost, easy to operate, and responsive to temperature changes, which is essential for handling minute samples. A characteristic feature of this method is the ability to easily cool samples by using glass capillaries or a space created by sandwiching a 20 µm thick spacer (shim ring) between cover glasses as micro-space sample containers, as well as through-holes in an electronic circuit board as macro-space sample containers. 

The results of tests utilizing these OSs revealed that *C. reinhardtii*, when exposed to low temperatures, nearly loses translational motility observed at room temperature, with most cells showing little to no change in central position and an increased number of cells exhibiting vibrational motility in place. Given that there are no reports of *C. reinhardtii* possessing specific sensory organs or taxis for temperature, these unique motility changes under low-temperature conditions are considered to be a deviation from the normal operation of flagellar motility.

As *C. reinhardtii* is highly responsive to light, the effect of light wavelength on cell motility under low-temperature conditions was investigated using four different types of LED light: white, red, green, and blue. Additionally, without physiological stressors such as low temperature, the analysis of cell motility in high-viscosity culture media at room temperature was conducted to understand the conditions under which cell swimming velocity and flagellar beat frequency decrease.

This study aims to report, for the first time, the unique motility of *C. reinhardtii* at low temperatures, which has not been previously investigated, and investigate its basic characteristics of motility through the aforementioned tests. This is an important discovery for deepening our understanding of cold stress responses and flagellar motility. It is expected to contribute to unraveling the strategies that *C. reinhardtii* employs to cope with cold conditions in the natural environment.

## 2. Materials and Methods

### 2.1. Sample Preparation

The wild-type *Chlamydomonas reinhardtii* CGC CC-125 mt^+^ (also known as 137c mt^+^ or IAM C-541) was used as a sample. *C. reinhardtii* was aerobically cultured in stationary conditions in TAP (Tris-Acetate-Phosphate) medium [29], under continuous illumination (white fluorescent light, 40 μmol photons/m^2^/s) at a temperature of 20 to 23 °C. For the experiments, cells (cell suspensions) from the logarithmic growth phase to the early stationary phase were used as samples.

### 2.2. Observation System

In this study, experiments were conducted using four observation systems (OS), with each having distinct characteristics. Table 1 provides an overview of each OS.

**Figure 1:**
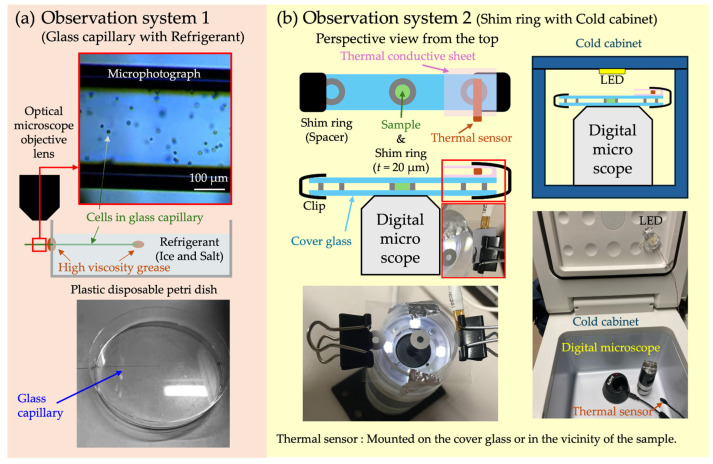
Observation Systems 1 and 2. (**a**) In OS 1, a mixture of ice and salt was used as the coolant, with a glass capillary serving as the sample containers. The end of the capillary was sealed with high-viscosity grease, and the sample was cooled by the refrigerant added to the petri dish. Cells within the capillaries, which protrude outside the dish, were observed using an optical microscope. (**b**) In OS 2, a cold cabinet was used, and a micro-well created with a shim ring (inner diameter Φ 2 mm, thickness 20 µm) served as the sample containers. This micro-well was formed by sandwiching a shim ring between two cover glasses. Samples fixed to a digital microscope were observed under a white LED installed on the ceiling of the cold cabinet.

In the OS 1 (Figure 1a), a refrigerant (a mixture of ice and salt) was used for cooling. The sample container was a glass capillary with an inner diameter of approximately Φ 300 µm. This capillary was created by heating and stretching a Pasteur pipette, after which the cell suspension was injected through capillary action. For observation, a small hole was drilled in the side of a plastic disposable petri dish, through which the capillary was inserted. The gap between the capillary and the hole was sealed with high-viscosity grease (MOLYKOTE^®^ High Vacuum Grease FS-50, TORAY INDUSTRIES, Inc., Tokyo, Japan), ensuring that the capillary was held horizontally. The end of the capillary inside the petri dish was also sealed with the same high-viscosity grease. This prevented the entry of the refrigerant into the capillary. The harmlessness of this high-viscosity grease to the cells was confirmed via preliminary tests. The refrigerant was added to the petri dish, serving to maintain the sample at a low temperature near 0 °C. An optical microscope (GR-D8T2, Shodensha Inc., Osaka, Japan) was used to observe the motility of cells inside the glass capillary protruding from the petri dish (Video S1). Objective lenses with magnifications of 10× (NA 0.25, Shodensha Inc., Osaka, Japan) and 40× (NA 0.65, Shodensha Inc., Osaka, Japan) were utilized. The hallmark of this system is its ease of operation and low cost. 

In OS 2 (Figure 1b), a cold cabinet capable of temperature adjustment (−20~20 °C, VS-CB009WH, VERSOS Co., Ltd., Hiroshima, Japan) was used. For sample container, a micro-well (diameter Φ 2 mm, thickness 20 µm) was created by sandwiching a shim ring (internal diameter Φ 2 mm, external diameter Φ 6 mm, thickness 20 µm, material SUS304, IWATA MFG. Co., Ltd., Gifu, Japan) between two cover glasses. Details of this method can be found in reference [15]. For observation, the sample was fixed to a wireless digital microscope (500×, 3R-WM601PC, 3R SYSTEMS CORP., Fukuoka, Japan) with double-sided tape. A white LED mounted on the ceiling of the cold cabinet served as the light source for observation, with the sample and microscope placed directly underneath. The temperature sensor was attached to the surface of the slide glass or positioned close to the sample. The temperature inside the cold cabinet gradually decreased from room temperature. During this period, the changes in temperature and the state of cell motility were recorded (Video S2). This method allows for the observation of cells in micro-spaces, and by setting up multiple microscopes, simultaneous measurements of different samples can be conducted.

**Figure 2:**
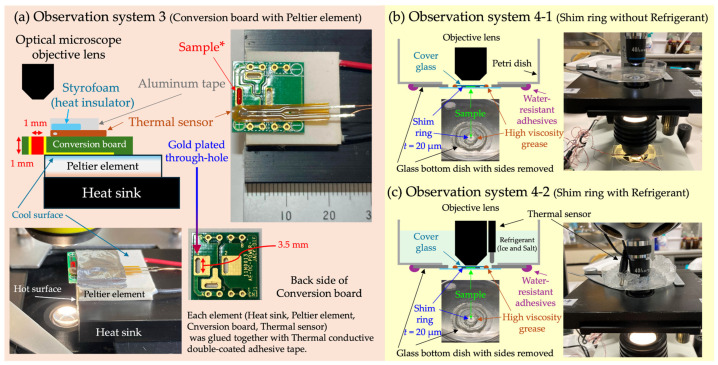
Observation Systems 3 and 4. (**a**) In OS 3, a conversion circuit board was fixed to the cooling side of the Peltier element. The through-hole of the conversion board, made of high-thermal-conductivity metal, serves as a temperature-adjustable sample container. A temperature sensor was fixed on the conversion board near the sample and secured with aluminum tape over insulating material. Cells within the through-hole were observed using an optical microscope. (**b**) In OS 4-1, a micro-well formed by placing a shim ring (inner diameter Φ 2 mm, thickness 20 µm) between the bottom of a glass-bottom dish with its sides removed, and a cover glass was used as the sample container. The gap between the glass-bottom dish and the cover glass was sealed with high-viscosity grease, preventing sample evaporation. A hole slightly smaller than the glass-bottom dish was made in the bottom of the petri dish, and the glass-bottom dish was adhered from underneath. Samples within the micro-well were observed using an optical microscope. (**c**) OS 4-2 allows for the observation of samples in a microspace under cold conditions by adding a refrigerant (ice and salt) to the petri dish used in OS 4-1. * “Sample” in (**a**): to enhance visibility, a solution of *Monascus* pigment was used instead of a cell suspension.

In OS 3 (Figure 2a), a cooling device combining a Peltier element (3.8 A, 20 × 20 mm, TEC1-03103-T100-SS-TF01-ALO, AKIZUKI DENSHI TSUSHO Co., Ltd., Tokyo, Japan) and a conversion circuit board for electronic components (DC jack DIP conversion board, AE-DC-POWER-JACK-DIP, AKIZUKI DENSHI TSUSHO Co., Ltd., Tokyo, Japan) was used. The through-hole of this conversion board (1.0 × 3.5 × 1.0 mm^3^) was made of metal with high thermal conductivity and was gold-plated, rendering it non-toxic to cells and able to function as a temperature-adjustable sample container. In Figure 2a, to enhance visibility, a solution of *Monascus* pigment was used instead of a cell suspension. *Monascus* pigment, derived from the filamentous fungus *Monascus* sp., serves as both a food coloring agent and a non-invasive pigment for cell viability assay [45,46,47]. The cooling side of the Peltier element is attached to the conversion board, while the heat-generating side is affixed to a heat sink via Thermal Conductive Double-Coated Adhesive Tape (TCATT). Additionally, a temperature sensor was fixed near the through-hole of the conversion board where the sample was injected using TCATT. The top surface of the temperature sensor was covered with styrofoam as insulation, which was secured with aluminum tape. The sample within the through-hole was observed using an optical microscope with a 40× objective lens (NA 0.65). The characteristic feature of this OS is its high controllability and responsiveness of temperature.

In OS 4 (Figure 2b,c), a refrigerant similar to that used in OS 2 was employed as the cooling method. The sample container was created by placing a shim ring, identical to the one used in OS 2, on the bottom surface of a glass-bottom dish (Matsunami glass D11130H, Matsunami Glass Ind., Ltd., Osaka, Japan) with its sides removed and then layering a cover glass on top. The gap between the cover glass and the bottom surface of the glass-bottom dish was sealed with high-viscosity grease. This serves to prevent sample evaporation in OS 4-1 and block the refrigerant from infiltrating the sample through the gaps around the shim ring in OS 4-2. A hole, slightly smaller than the glass-bottom dish, was made in the bottom of the plastic disposable petri dish, and the glass-bottom dish was glued from underneath with waterproof adhesive, ensuring the sample surface faced upwards. A temperature sensor was placed near the sample. The motility of cells, either with added refrigerant or without, within this petri dish was observed using an optical microscope with a 40× objective lens (NA 0.65). The main feature of OS 4 is its ability to observe cells more affordably compared to the method using a cold cabinet in OS 2.

### 2.3. Quantification of Cell Center Trajectories

In each OS, the motility of cells was observed at room temperature and under cold conditions, and the process was recorded as video files by a recording device. As recording devices, either a wireless digital microscope or a high-speed camera (CHU30-C-RS, Shodensha Inc., Osaka, Japan) was mounted on the optical microscope in place of the eyepiece lens.

The analysis of the obtained video files was carried out as follows: Firstly, it was confirmed that the videos were in AVI format, and they were imported into ImageJ. Next, the videos were converted to black and white images using the “Convert to Grayscale” function, and the area for analysis was selected and cropped using the rectangle tool. The “Subtract Background” function was used to eliminate the brightness gradient of the background, and the “Threshold” function was employed to binarize the images, adjusting the threshold as necessary. To enhance the visibility of *C. reinhardtii*, the “Black background (of binary masks)” was used to invert the black and white of the binarized images. The “Median” filter was applied to remove noise, and the “Scale Bar” function was used to insert a scale bar into the image. The “Particle Tracker 2D/3D” plugin saved the tracking data of the *C. reinhardtii* cell center coordinates. The tracking data included the “number of identified cells”, “frame number in which the cell is present”, and “two-dimensional coordinates of the cell center. 

### 2.4. Analysis of Cell Swimming Velocity

In the analysis of cell center velocity, we first selected the most suitable period for analysis from the recorded video files. This period was chosen based on the clarity of cell motility and the continuity of such motility under constant conditions. Next, using the first frame of the video from the selected period as a reference, the position coordinates of each cell center were identified. This initial position served as a reference point for tracking changes in cell location in subsequent frames. For each frame of the video, the position coordinates of the cell center were identified, and the relative distance from each reference point was calculated. This distance measurement was conducted to quantify the path traversed by the cells.

Using the obtained distance data and the video’s fps (frames per second), the velocity of each cell was calculated. This allowed an average of the velocities of all cells in the observation area to be calculated. 

### 2.5. FFT Analysis of Cell Motility

The swimming motility of *C. reinhardtii* is characterized by cyclic motility, driven by the repetitive effective and recovery strokes of its flagella. In this study, we examined how this cyclic motility differs between room temperature and low temperature, and whether there are differences in its regularity. The analysis procedure is as follows:

Firstly, from the recorded videos, a suitable time period for analysis and one cell within that time period were selected. This selection aimed to identify the time and cell where the cyclic motility was most prominently observed. In the chosen observation period, the position coordinates of the cell center in the first frame were used as a reference point. Subsequently, the distance from this reference point to the cell center’s position coordinates in each frame was calculated. This distance serves as an indicator of how much the cell has moved. 

Next, the relationship between the distance of the cell center from the reference point and the elapsed time over the observation period was graphed (Figures S1b–S10b, black line). For this graph, a fifth-degree polynomial curve fit was performed (Figures S1b–S10b, red line). The fifth-degree polynomial curve was subtracted from the measured data, and the temporal displacement of the cell center’s oscillation was graphed (Figures S1c–S10c).

Finally, a home-made Fortran program was used to perform fast Fourier transform (FFT) analysis of the obtained data, mathematically extracting periodic components. It should be noted that the wireless digital microscope has a frame rate of 30 fps, which is lower than the flagellar beat frequency of *C. reinhardtii* (60 Hz); hence, it was used for analyzing the swimming velocity of cells. On the other hand, the frame rate of the high-speed camera, capable of up to 1000 fps, was used for analyzing the oscillations of the cell center.

### 2.6. Relationship between Cell Swimming Velocity and Temperature

**Figure 3:**
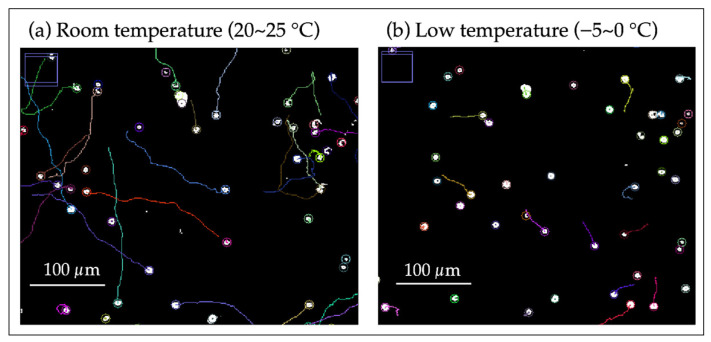
Trajectory of cell center at room temperature and low temperature: (**a**) at room temperature; (**b**) at low temperature These images show the trajectory of the cell center for approximately 3.3 s (30 fps, 100 frames) and were created from video data at any given time in each temperature range. The color of the trajectory corresponds to each cell.

**Figure 4:**
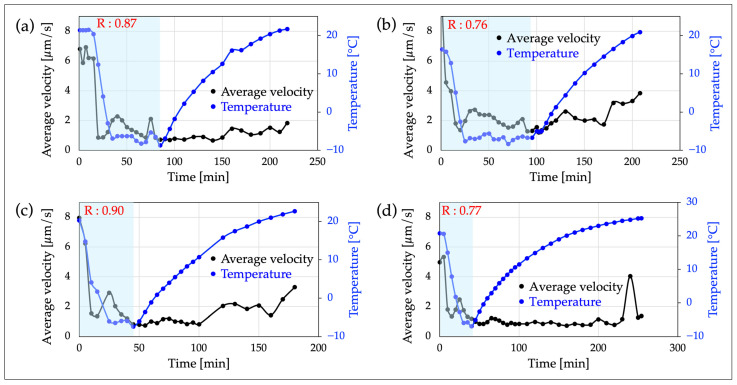
Relationship between the average velocity of cells and temperature: (**a**–**d**) are the results of experiments with identical content using different samples. The light blue highlighted areas in these graphs represent the period from when the temperature of the experimental environment starts to drop from room temperature, remains below freezing for a certain period, and then begins to rise again. Additionally, “R” indicates the value of the correlation coefficient between average velocity and temperature during this light blue highlighted period. In all results, R is greater than 0.76, so there is a positive correlation between temperature and mean swimming speed.

In OS 2, the relationship between swimming velocity and temperature of C. reinhardtii was investigated. Figure 3 shows the trajectory of the cell center’s motility over approximately 3.3 s (30 fps, 100 frames) under room and cold temperature conditions. In this experiment, the temperature was gradually lowered from room temperature to below 0 °C using a cold cabinet and maintained at that temperature range for a certain period, and then the temperature was raised again. During this observation period, the relationship between the average velocity of the cells present in the observation area and the temperature was graphed (Figure 4).

For the creation of the graph, data from 30 fps and 100 frames (approximately 3.3 s) were used for velocity analysis at each elapsed time, plotting the average velocities of cells and temperature every 3–10 min. Furthermore, the correlation coefficient between the temperatures and average velocities of cells were calculated for the period from the start of the observation until just before the temperature was raised again (Figure 4). 

The recording device used for this series of measurements was a wireless microscope (resolution: 640 × 480, Frame rate: 30 fps). Similar tests were conducted four times using different samples. Video S2 shows the motility of cells during the cooling process from room temperature to below 0 °C in OS 2. 

### 2.7. FFT Analysis of Cell Motility with Respect to Temperature and Wavelength

**Figure 5:**
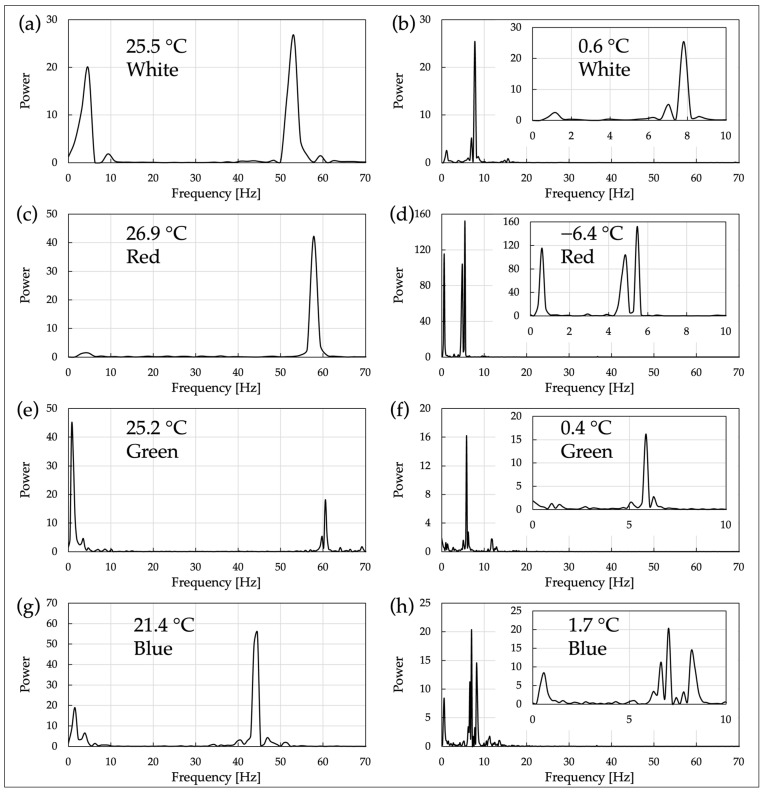
FFT analysis of cell motility by temperature and irradiation light wavelength. (**a**–**h**) Each graph includes the temperature and the color of the observation light LED. The central wavelength of each LED is referred to in Table 2. (**c**) Due to missing frames in the original data used for FFT analysis, interpolation was performed by averaging the values before and after (number of interpolated frames: 1; number of data used in FFT analysis: 128).

In OS 3, the motility of C. reinhardtii was measured under two temperature conditions: room temperature condition and low-temperature condition. For each temperature condition, cell motility was observed using an optical microscope equipped with power LEDs of white, red, green, and blue as observation light sources. A high-speed camera (resolution: 640 × 380, Frame rate: 200 fps) and an optical microscope with a 40× objective lens (NA 0.65) were used as recording devices. The obtained video data were processed through FFT analysis to quantify the motility characteristics of the cells and graphed (Figure 5 and Figures S1d–S10d).

### 2.8. FFT Analysis of Cell Motility in Viscous Solution

In OS 4, the motility of *C. reinhardtii* under different viscosity conditions was analyzed. Measurements were conducted on two types of samples: a cell suspension in normal TAP medium (OS 4-1) and a cell suspension in TAP medium with the concentration of Ficoll (Ficoll^®^ PM 400, Sigma-Aldrich, St. Louis, MO, USA) adjusted to 7% (*w/w*) (OS 4-2). Ficoll is a highly branched polymer produced through the copolymerization of sucrose and epichlorohydrin. Ficoll, being completely nonionic and hydrophilic due to its abundance of hydroxyl groups, exhibits high water solubility. Its large molecular weight significantly reduces permeability through the cell membrane compared to sucrose, thus maintaining cellular functions and morphology more effectively due to its low membrane permeability and osmotic properties [48].

**Figure 6:**
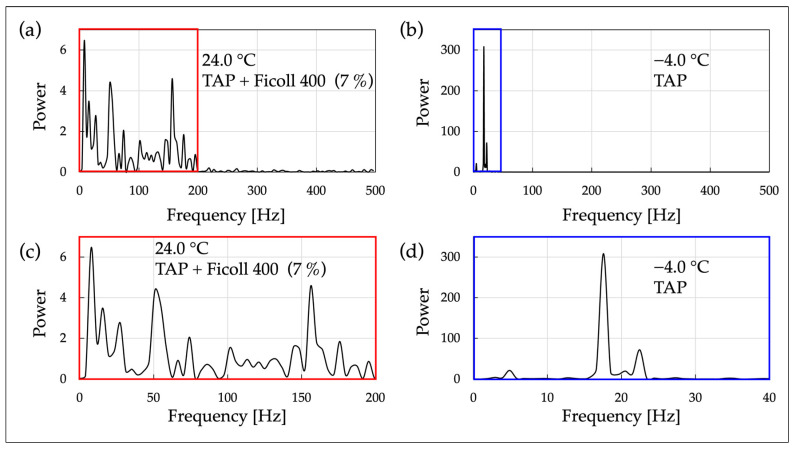
FFT analysis of cell motility in culture media with different temperatures and viscosities: (**a**) motility in high-viscosity TAP medium at room temperature; (**b**) FFT analysis of cell motility in TAP medium under cold conditions; (**c**) a magnified view of the prominent frequency range of peak structures in (**a**); (**d**) a magnified view of the prominent frequency range of peak structures in (**b**). (**a**,**b**) Due to missing frames in the original data used for FFT analysis, interpolation was performed by averaging the values before and after ((**a**) Number of interpolated frames: 2; number of data used in FFT analysis: 128). ((**b**) Number of interpolated frames: 2; number of data used in FFT analysis: 1024).

Regarding temperature conditions, the OS 4-1 samples were measured at room temperature, while the OS 4-2 samples were measured under cold conditions. In each experiment, frequency analysis was conducted on an individual cell. A high-speed camera (resolution: 640 × 380, frame rate: 1000 fps) and an optical microscope with a 40× objective lens (NA 0.65) were used as recording devices. The obtained video data were processed using FFT analysis to extract the periodic motility patterns of the cells, and the results are presented in Figure 6.

Regarding sample preparation, the method used for adjusting the solution to dissolve Ficoll at a 7% concentration in the cell suspension was as follows: Firstly, Ficoll was dissolved in fresh TAP medium to adjust it to a concentration higher than the desired 7%. This high-concentration Ficoll solution was then mixed with the cell suspension at a specified ratio to create a cell suspension with the resulting required Ficoll concentration.

The conditions for each of the above experiments are presented in Table 2.

**Table 2:** Measurement conditions for each experiment.

Section	Temperature (°C)	Observation Light Source (Center Wavelength (nm))	Photon Flux Density (μmol Photons/m^2^/s)	Frame Rate (fps)	Resolution	Recording Device	Concentration of Ficoll (%(*w*/*w*))	Observation System
Section 3.1 Video S1	-	Halogen lamp (-)	-	30	560 × 336 (Original data)	iPhone SE	0	1
Section 2.6 Section 3.1	Room temperature ~ Low temperature	White LED (-)	-	30	640 × 480	Wireless digital microscope		2
Section 2.7 Section 3.2	25.5	White LED (-)	10	200	640 × 380	High-speed camera and optical microscope with a 40× objective lens (NA 0.65)		3
	0.6							
	26.9	Red LED (625)	24					
	−6.4							
	25.2	Green LED (525)	23					
	0.4							
	21.4	Blue LED (470)	46					
	1.7							
Section 2.8 Section 3.3	−4.0	Halogen lamp (-)	95	1000				4
	24.0						7	

## 3. Results

### 3.1. Relationship between Cell Swimming Velocity and Temperature

Initially, the simplest experimental setup, OS 1, confirmed that C. reinhardtii exhibits a different motility at low temperatures (significant reduction in swimming speed and pronounced oscillatory movements) compared to room temperature (Video S1). To investigate the motility in greater detail, subsequent tests employed setups OS 2 to OS 4 as appropriate.

Measurements of cell motility by OS 2 showed the trajectories of cells at room and low temperatures (Figure 3) and the relationships between the average swimming velocity of cells within the observation range and temperature (Figure 4). 

Figure 3 shows that the swimming distance of cells decreased at the same time, and the number of cells oscillating in situ increased under low-temperature conditions compared to room temperature.

From Figure 4, it can be seen that as the temperature decreases, the average swimming velocity of the cells decreases, and a low velocity is maintained during the period when the temperature is kept below freezing. When the temperature begins to rise, there is a tendency for the swimming velocity of the cells to gradually recover. However, the velocity increase is small. 

The correlation coefficient “R” listed in each graph represents the strength of the correlation between temperature and average swimming velocity during the light blue highlighted part (the period when the temperature drops from room temperature, maintains a certain temperature below freezing, and then rises again), and it was above 0.76. This indicates a highly reproducible and positive correlation between temperature and the mean swimming speed. It should be noted that the reason the initial average velocity is lower than the typical velocity of *C. reinhardtii* is that the cells are placed in a two-dimensional microspace (Φ 2 mm × 20 µm) and include a large number of stationary cells (Figure S11).

### 3.2. FFT Analysis of Cell Motility with Respect to Temperature and Wavelength

*C. reinhardtii* is highly responsive to light, exhibiting behaviors such as phototaxis. Therefore, we investigated whether its motility at low temperatures is dependent on the wavelength of the light used for irradiation. Based on the results obtained from OS 3, the oscillatory motility of a specific single cell under room temperature and cold conditions was analyzed using FFT analysis. Here, a different LEDs of a different wavelength was used as the observation light source under each temperature condition (Table 2). The results are shown in Figure 5.

Figure 5 shows the FFT analysis results of the motility of *C. reinhardtii* under different irradiation light wavelengths and temperature conditions. Each graph represents the frequency response of cell motility when exposed to light of a specific wavelength (white, red, green, blue) under different temperatures. (a), (c), (e), and (g) are the FFT analysis results when exposed to white, red, green, and blue wavelengths of light at room temperature (25.5 °C, 26.9 °C, 25.2 °C, 21.4 °C), respectively, with major peaks observed at 45~61 Hz. On the other hand, (b), (d), (f), and (h) are the analysis results under cold conditions (0.6 °C, −6.4 °C, 0.4 °C, 1.7 °C), showing changes in peak frequency compared to room temperature. Notably, under cold conditions, all samples show major peaks in the lower frequency band (5~10 Hz), indicating that the stimulus from cold temperatures has a more significant impact on the motility pattern of cells than the wavelength of irradiation light.

### 3.3. FFT Analysis of Cell Motility in Viscous Solution

Based on the results obtained from OS 4, FFT analysis was performed, focusing on one specific cell for each cell motility in TAP medium at low temperature and in viscous solution at room temperature (Figure 6). 

From Figure 6a, it is observed that the FFT spectrum of cell motility in a culture medium with high viscosity (3.5 mPa·s) at room temperature contains multiple peaks. However, the increase in the number of peaks compared to that under cold conditions in Figure 5, and their occurrence across a broader frequency band (approximately 15 peaks within the range of 0~200 Hz), suggests an increase in the irregularity of cell motility. 

From Figure 6b, it is clear that cell motility in a non-viscous solution under cold conditions has distinct peaks at specific frequencies, showing a similar trend to the results under cold conditions in Figure 5. The difference between Figure 5 and Figure 6 lies in the scale of containment; cells are contained within a macro-scale container (1.0 × 3.5 × 1.0 mm^3^) in Figure 5, whereas in Figure 6, they are contained within a micro-scale container (Φ 2 mm × 20 µm). 

## 4. Discussion

In this study, observations and measurements using four different OSs were conducted to analyze the motility of *C. reinhardtii* under cold conditions (Figure 1 and Figure 2, Table 1). Through these OSs, characteristic patterns of cell motility were confirmed at temperatures around 0 °C or below. Specifically, notable decreases in the swimming velocity and oscillation frequency of cells compared to room temperature were observed across all OSs.

From Figure 3, it was confirmed that under cold conditions compared to room temperature, the swimming distance of cells within the same time period significantly decreased, and there was a tendency for an increase in the number of cells oscillating in place. This suggests that the decrease in temperature affects the energy dynamics of cell motility, directly imposing constraints on the ability to move. Cells not swimming at room temperature include those adhered to the cover glass, those temporarily immobilized due to the impact at the time of encapsulation, or dead cells (Figure 3a).

From Figure 4, in all four similar experiments, it was observed that the average swimming velocity of *C. reinhardtii* decreases as the temperature drops from room temperature, and this reduced velocity is maintained during the period when the temperature is kept below freezing. Moreover, as the temperature begins to rise, there as a tendency for the swimming velocity of the cells to gradually recover, but this increase in velocity was shown to be very minimal. This suggests that the cold stress had a certain impact on the cells, potentially causing irreversible damage.

The correlation coefficient between temperature and average swimming speed during the light blue highlighted period in Figure 4 (from the start of measurement, as the temperature drops from room temperature and then rises again) shows values above 0.76 in all OSs, with an average value of 0.82 from four experiments. This implies that the decrease in temperature and the decrease in cell swimming velocity are roughly synchronized. Similar trends were obtained in all four tests, confirming their high reproducibility.

Additionally, the lower average velocity at the start of the measurement at room temperature compared to the typical swimming velocity of *C. reinhardtii* might be due to the cells being observed in a two-dimensional microspace. Cells that cannot swim due to their flagella adhering to the top or bottom of cover glasses, cells that temporarily become immobile due to the pressure during shim ring encapsulation, and dead cells are recognized as stationary cells, which could be factors contributing to the lower average velocity. For Figure 4a, histograms of speeds at each elapsed time are shown in Figure S11. From this, it is evident that at all times, there were many cells with velocities near zero. 

From Figure 5, distinct major peaks were observed in the FFT spectrum under room temperature conditions within the range of 45–61 Hz. This indicates that the swimming motility of *C. reinhardtii* includes regular oscillatory components predominantly at these frequencies. In contrast, under cold conditions, distinct major peaks appeared at a lower frequency range (5–10 Hz) compared to room temperature, indicating that the oscillations retain a degree of regularity similar to those at room temperature. This reveals that the characteristic of swimming motility at low temperatures includes oscillatory components at lower frequencies. This can be confirmed by lowering the temperature, which allows the cells to visibly vibrate under the microscope (Videos S1 and S2).

The presence of distinct major peaks in the FFT spectrum at 45–61 Hz at room temperature is thought to reflect the flagellar beat frequency of *C. reinhardtii*, which is around 60 Hz. This suggests that the reduction in the major peak frequency to 5–10 Hz in the FFT spectrum under cold conditions may indicate that the flagellar beat frequency also decreases due to the drop in temperature. This decrease in frequency could be due to the suppression of enzymatic reactions governing flagellar motility by the low temperature, hindering normal flagellar motility. However, the results of the FFT analysis at low temperatures, with a few clear peaks at specific frequency bands, suggest that the regularity of flagellar motility is maintained. 

**Figure A1:**
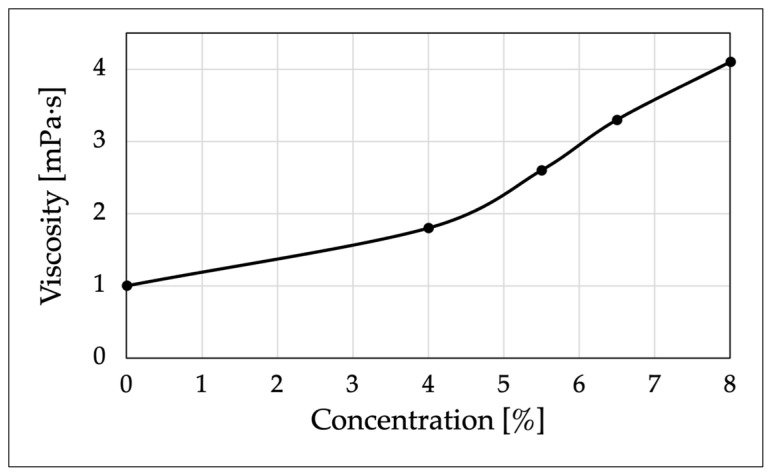
Relationship between concentration and viscosity of Ficoll 400; edited based on [42].

Thus, to induce a significant change in the state of cell motility without the influence of temperature (suppression due to low temperatures) on the enzymes governing flagellar motility, we considered increasing the viscosity of the cell suspension at room temperature. Specifically, the viscosity of the cell suspension was adjusted using Ficoll, which exhibits Newtonian fluid properties. According to previous studies [49], under conditions where the viscosity of the Ficoll solution is 3.5 mPa·s, the flagellar beat frequency decreases by about 30% compared to that in a non-viscous solution, and the swimming velocity decreases by 40%. Also, no significant decrease in flagellar beat frequency was observed under conditions with viscosities lower than this value. Therefore, based on the relationship between the Ficoll concentration and viscosity documented in reference [42] (Figure A1), the Ficoll concentration was set to 7%, which corresponds to a viscosity of 3.5 mPa·s. Moreover, it has previously been confirmed that the differences in viscosity and density between water (H_2_O, a typical calibration liquid) and TAP medium can be disregarded in the frequency response of *C. reinhardtii* flagella [50].

From Figure 6a, the FFT spectrum of cell motility in high-viscosity solution at room temperature exhibited significantly more peaks over a broader frequency range (approximately 15 peaks within the range of 0~200 Hz) compared to the non-viscous solution at low temperature (Figure 6b). This indicates that the irregularity of the oscillatory components of cell motility is increased in high-viscosity solution compared to non-viscous solution. Therefore, as conditions that clearly change the nature of cell motility, there are two scenarios: a viscous solution at room temperature and a non-viscous solution at low temperature. However, from the perspective of oscillatory regularity, it has been found that these conditions differ. 

From Figure 6b, the FFT spectrum of cell motility in non-viscous solution at low temperatures shows a distinct major peak at a lower frequency (18 Hz), which is in line with the results under cold conditions seen in Figure 5. In Figure 5, cells were contained in the through-hole of a conversion circuit board (1.0 × 3.5 × 1.0 mm^3^), whereas in Figure 6, they are confined within a two-dimensional microspace (Φ 2 mm × 20 µm). This indicates that the oscillatory motility of cells at low temperatures does not depend on the scale of the space surrounding the cells.

**Figure A2:**
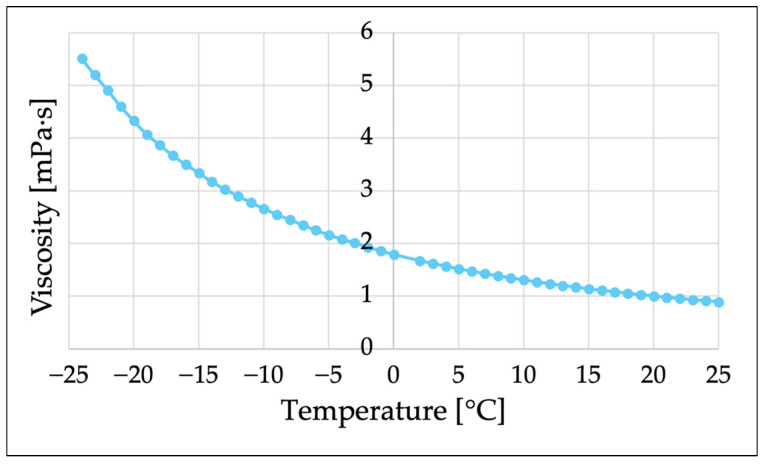
Relationship between water temperature and viscosity; data below 0 °C were compiled based on [52], and other data were compiled based on [53].

Water viscosity increases as the temperature decreases; so, to achieve the viscosity (3.5 mPa·s) of the 7% Ficoll solution used in this experiment by cooling, it would be necessary to lower the temperature to about −16 °C (Figure A2). At the macroscale, water would freeze at this temperature, but at the microscale, it has been confirmed that a stable supercooled state can form [51]. In fact, in the test of Figure A2, the viscosity of supercooled water at −23.8 °C was measured using a capillary of Φ 200 µm × 150 mm [52]. It is conceivable that the motility of *C. reinhardtii* at such temperatures can be observed with the microspace measurement systems (OS 1, 2, 4) used in this study. However, this verification is beyond the scope of this paper and is positioned as a subject for future research.

## 5. Conclusions

In this study, to elucidate the motility characteristics of *C. reinhardtii* at lower temperatures (−8.7 °C to 1.7 °C) compared to the 15 °C cold conditions previously investigated, experiments were conducted using four types of OSs. These OSs are all low in cost and easy to operate. Furthermore, three of these systems are expected to exploit the properties of the microspace to enable motility analysis under stable supercooled conditions at even lower temperatures.

The experimental results using these OSs showed that under cold conditions, there is a significant decrease in the swimming velocity and oscillation frequency of cells, indicating that direct constraints are placed on the energy dynamics of cell motility. FFT analysis of cell motility revealed that under room temperature conditions (20–27 °C), the major peak frequencies were in the range of 45–61 Hz. This is thought to reflect the flagellar beat frequency of approximately 60 Hz in *C. reinhardtii*. 

Under cold conditions, major peaks were observed in the range of 5–18 Hz, suggesting a reduction in the flagellar beat frequency. Both at room temperature and under cold conditions, the FFT spectra of cell motility consisted of a few clear peaks, indicating regular oscillations. This trend was independent of the wavelength of irradiation light and whether the space scale where the cells were placed was macro or micro.

Without being subjected to physiological factors such as cold stress, conditions leading to a decrease in cell swimming velocity and flagellar beat frequency were observed by examining cell movement in high-viscosity culture medium (3.5 mPa·s) adjusted with Ficoll, which exhibits Newtonian fluid characteristics, at room temperature. FFT analysis was conducted, and the results showed approximately 15 peaks within the frequency range of 0~200 Hz. This indicates that in viscous fluids, unlike the movement in non-viscous fluids at room and cold temperatures, the irregularity of the oscillatory components of cell motility increased. Additionally, the irregularity of flagellar motility in viscous solutions at room temperature indicates that the regular and beat-frequency-decreasing behavior of flagellar motility at low temperatures was not due to an increase in water viscosity caused by a decrease in temperature.

The results confirm that the motility of *C. reinhardtii* under cold conditions is unique. To elucidate the cause of this phenomenon, a detailed analysis of flagellar motility is required. Introducing phase-contrast microscopy into the OSs of this study will enable a more detailed analysis of flagellar motility under cold conditions. While this study used Newtonian fluids at room temperature, future work will also involve comparing motility in viscous solutions at room and cold temperatures using viscoelastic solutions like polyvinyl alcohol (PVA), as well as analyzing motility at temperatures even lower than those studied here. In particular, considering the inverse relationship between water’s temperature and viscosity, we aim to conduct more detailed analyses. The results of this study, along with these future endeavors, may contribute to our understanding of the motility of *C. reinhardtii* in response to temperature and viscosity, as well as elucidating the strategies that *C. reinhardtii* employs to cope with cold conditions in the natural world. 

## Figures and Tables

**Table 1:** Characteristics of Observation Systems.

Observation System (OS)	Cooling Method	Sample Container	Capacity	Features
1	Refrigerant	Glass capillary	Φ 300 µm × 50 mm	Easy operation and low cost
2	Cold cabinet	Sim ring	Φ 2 mm × 20 µm	Observations in microspace Multiple sample measurements
3	Peltier element	Conversion board	1.0 × 3.5 × 1.0 mm^3^	High controllability of temperature High responsiveness of temperature
4	Refrigerant	Sim ring	Φ 2 mm × 20 µm	Observations in microspace Less expensive than OS 2

## Data Availability

Data are contained within the article and its Supplementary Materials.

## Supplementary Materials

The following supporting information can be downloaded via this link: https://www.mdpi.com/article/10.3390/mi15030410/s1, Figure S1: Analysis of cell motility at room temperature (25.5 °C) using a white LED as a light source (Figure 5a); Figure S2: Analysis of cell motility at low temperature (0.6 °C) using a white LED as a light source (Figure 5b); Figure S3: Analysis of cell motility at room temperature (26.9 °C) using a red LED as a light source (Figure 5c); Figure S4: Analysis of cell motility at low temperature (−6.4 °C) using a red LED as a light source (Figure 5d); Figure S5: Analysis of cell motility at room temperature (25.2 °C) using a green LED as a light source (Figure 5e); Figure S6: Analysis of cell motility at low temperature (0.4 °C) using a green LED as a light source (Figure 5f); Figure S7: Analysis of cell motility at room temperature (21.4 °C) using a blue LED as a light source (Figure 5g); Figure S8: Analysis of cell motility at low temperature (1.7 °C) using a blue LED as a light source (Figure 5h); Figure S9: Analysis of cell motility in viscous solution (Ficoll 7%, 3.5 mPa·s) at room temperature (24.0 °C) using a halogen lamp as a light source (Figure 6a); Figure S10: Analysis of cell motility at low temperature (−4 °C) using a halogen lamp as a light source (Figure 6b); Figure S11: Histogram of average velocity of cells (Figure 4a); Video S1: Motility at room and low temperatures (OS 1); Video S2: Motility at various temperatures (OS 2).
