# Supplementary material for: Analysis of Unique Motility of the Unicellular Green Alga Chlamydomonas reinhardtii at Low Temperatures down to −8 °C
Source: Micromachines (Basel). 2024 Mar 19;15(3):410. doi: 10.3390/mi15030410 (PMC10972011; doi:10.3390/mi15030410)
Supplement: Supplementary file 1 [file micromachines-15-00410-s001.zip › micromachines-2900026-supplementary.pdf]

Supplementary information

Analysis of Unique Motility of the Unicellular Green Alga *Chlamydomonas reinhardtii* at Low Temperatures Down to −8°C

1. Motility analysis of *Chlamydomonas reinhardtii*

Figures S1 to S10 shown below provide detailed data on the following items in addition to the results of vibrational analysis (FFT) of *Chlamydomonas reinhardtii* covered in the main text (Figures 5 and 6).

1. Trajectory of cell motility: The trajectory of the central motility of the cell to be analyzed is shown. However, the trajectory is within an arbitrary observation time range.

2. Changes in the distance traveled by cells relative to the origin over time: The origin was the cell center position at the start of observation, and the relationship between the elapsed time and the distance between the origin and the cell was graphed.

3. Approximate curve in 2: The curve in 2 above was fitted with a 5th order function and shown in the same graph.

4. Difference graph between 2 and 3: This graph shows the difference between the actual motion data and the 5th order approximation curve.

5. FFT analysis: The FFT analysis of 4 is shown. The analysis results are shown in a wider frequency range than those published in the main text.

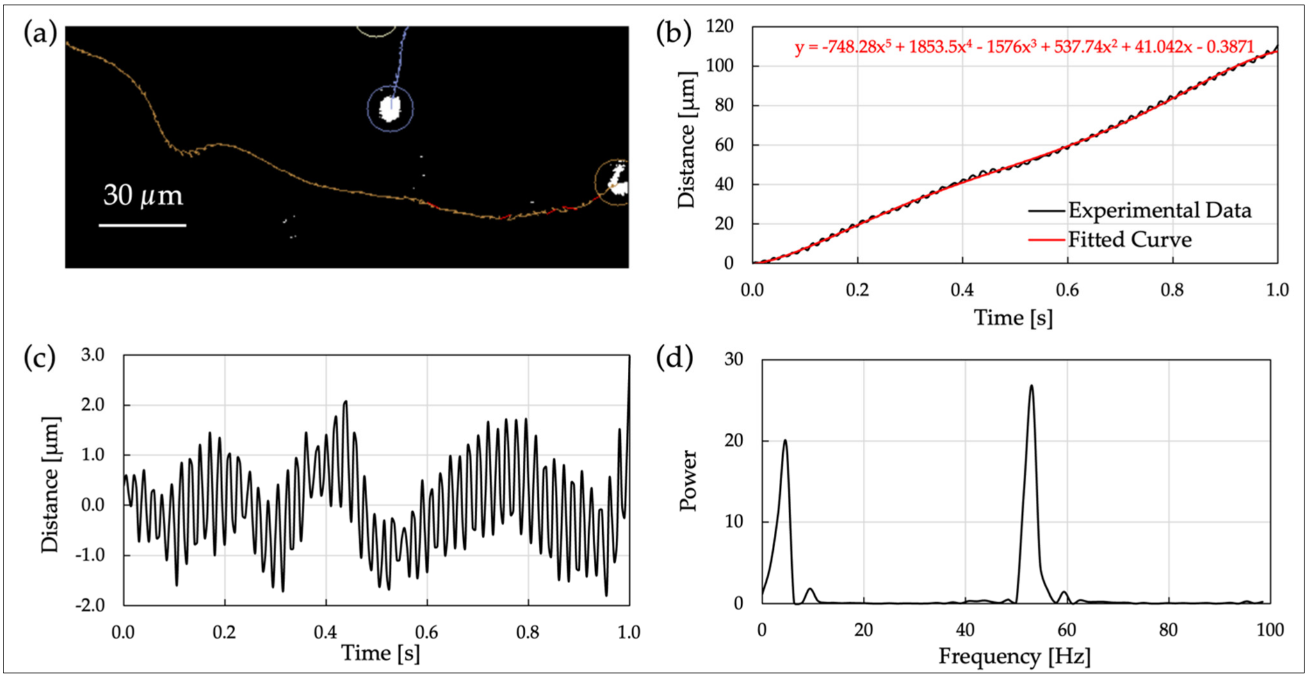

Figure S1. Analysis of cell motility at room temperature (25.5 °C) using a white LED as a light source (Figure 5a): (a) Trajectory of cell motility; (b) Change in cell swimming distance over time; (c) Difference between the measured value and the approximate curve in (b); (d) FFT analysis result of (c)

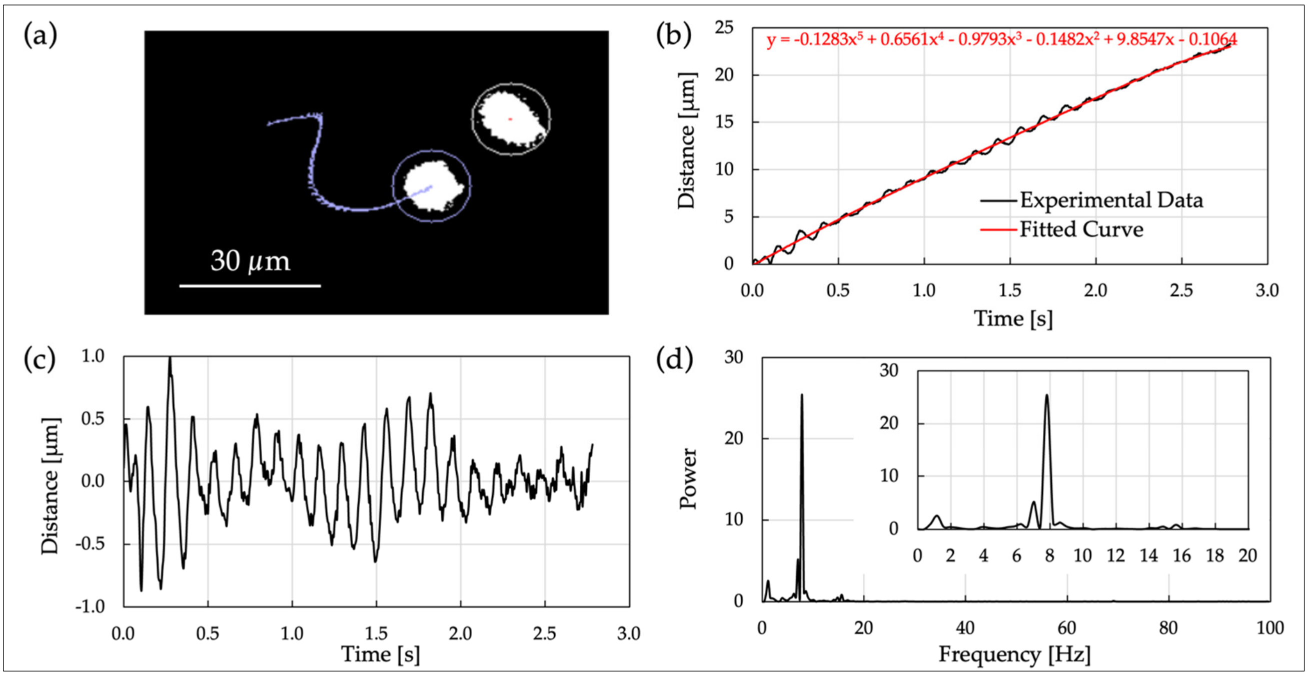

Figure S2. Analysis of cell motility at low temperature (0.6 °C) using a white LED as a light source (Figure 5b): (a) Trajectory of cell motility; (b) Change in cell swimming distance over time; (c) Difference between the measured value and the approximate curve in (b); (d) FFT analysis result of (c)

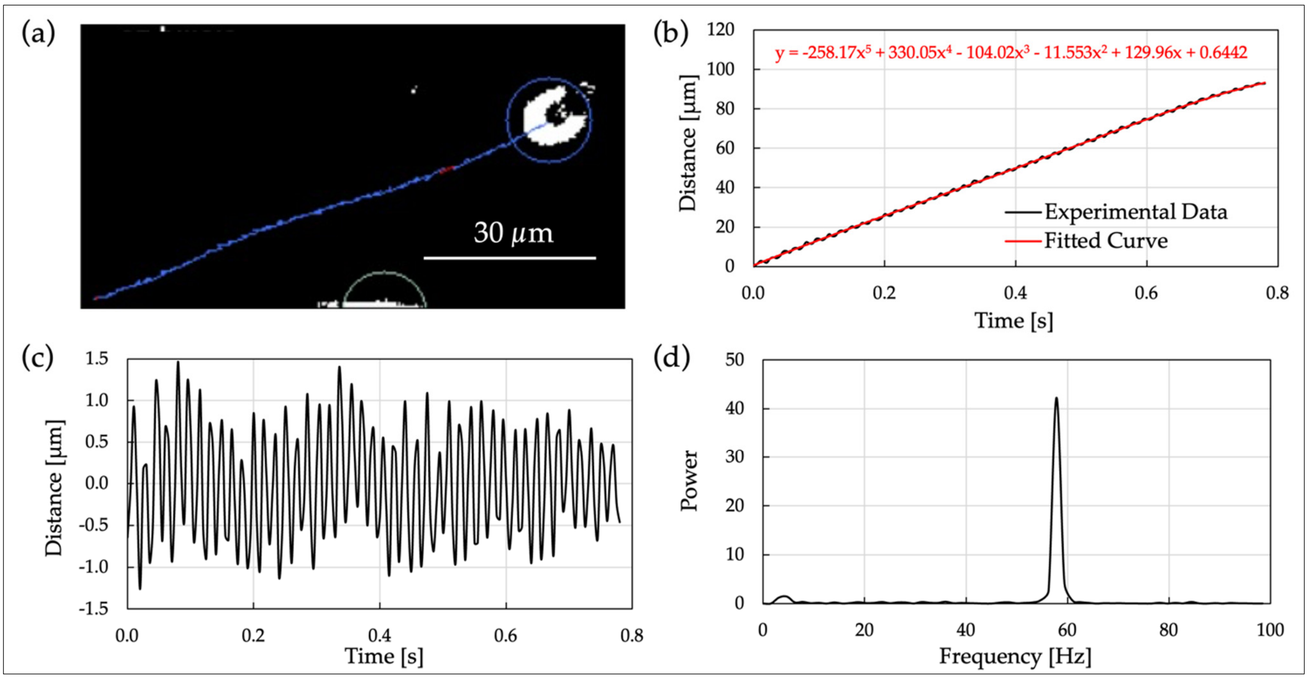

Figure S3. Analysis of cell motility at room temperature (26.9 °C) using a red LED as a light source (Figure 5c): (a) Trajectory of cell motility; (b) Change in cell swimming distance over time; (c) Difference between the measured value and the approximate curve in (b); (d) FFT analysis result of (c)

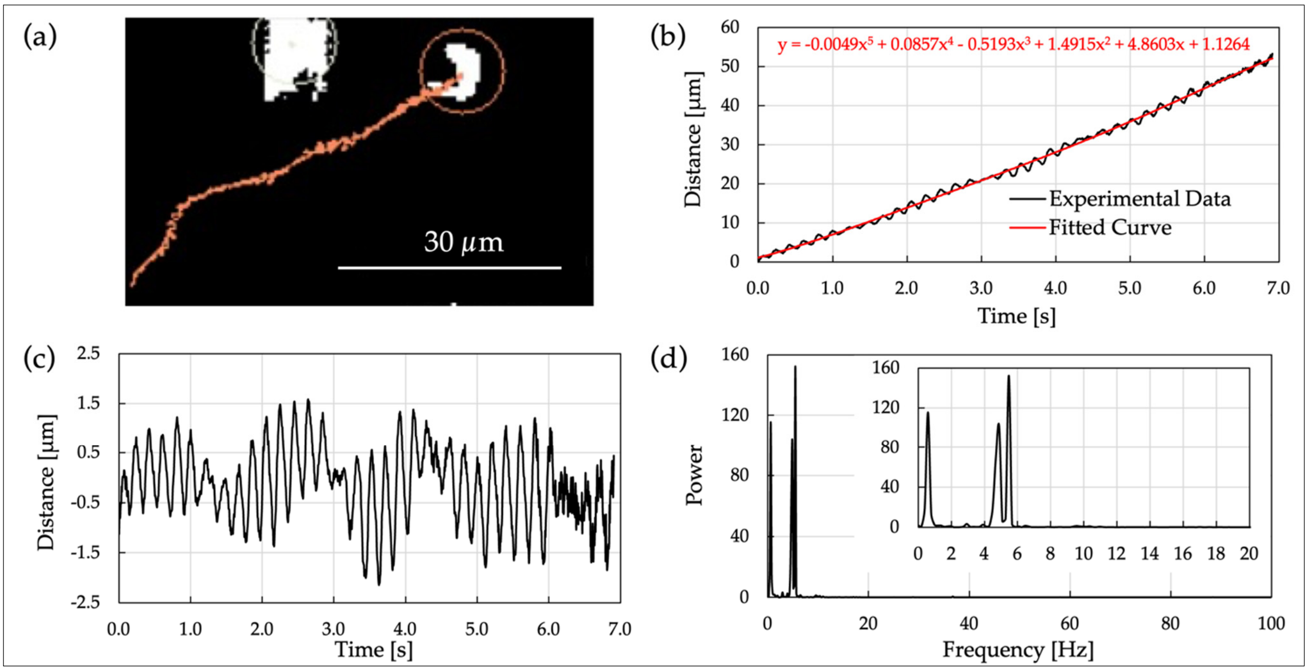

Figure S4. Analysis of cell motility at low temperature (6.4 °C) using a red LED as a light source (Figure 5d): (a) Trajectory of cell motility; (b) Change in cell swimming distance over time; (c) Difference between the measured value and the approximate curve in (b); (d) FFT analysis result of (c)

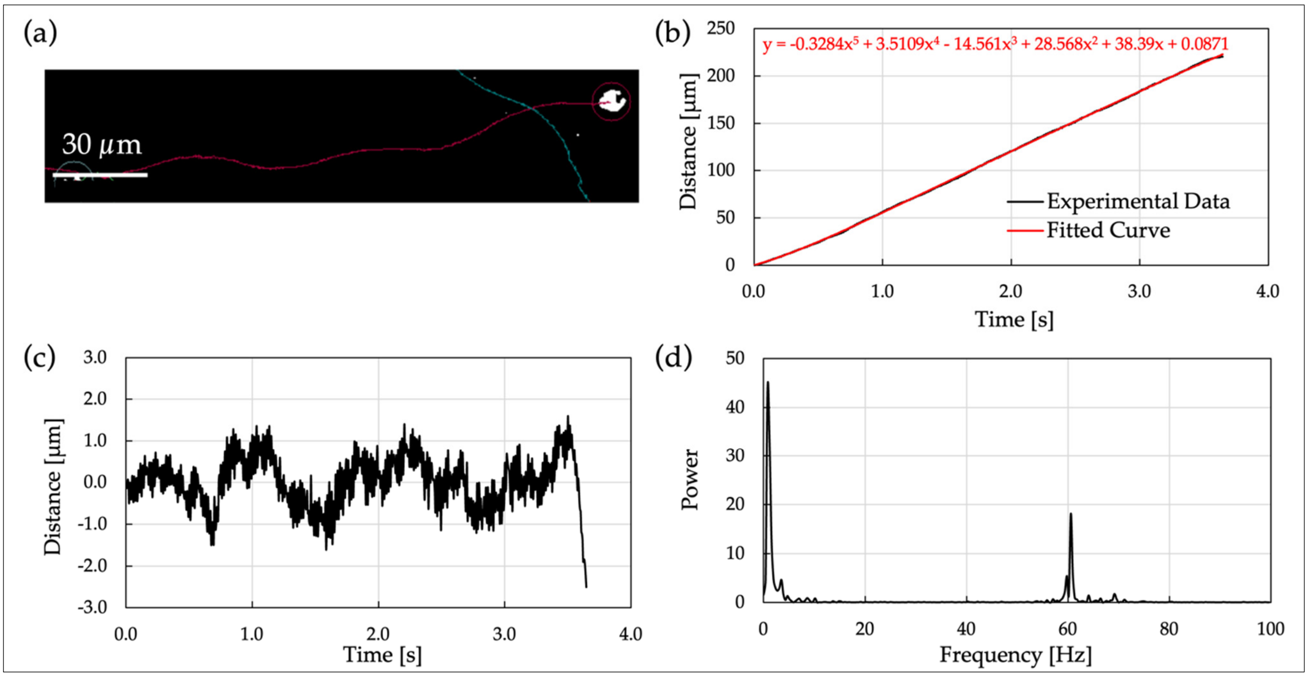

Figure S5. Analysis of cell motility at room temperature (25.2 °C) using a green LED as a light source (Figure 5e): (a) Trajectory of cell motility; (b) Change in cell swimming distance over time; (c) Difference between the measured value and the approximate curve in (b); (d) FFT analysis result of (c)

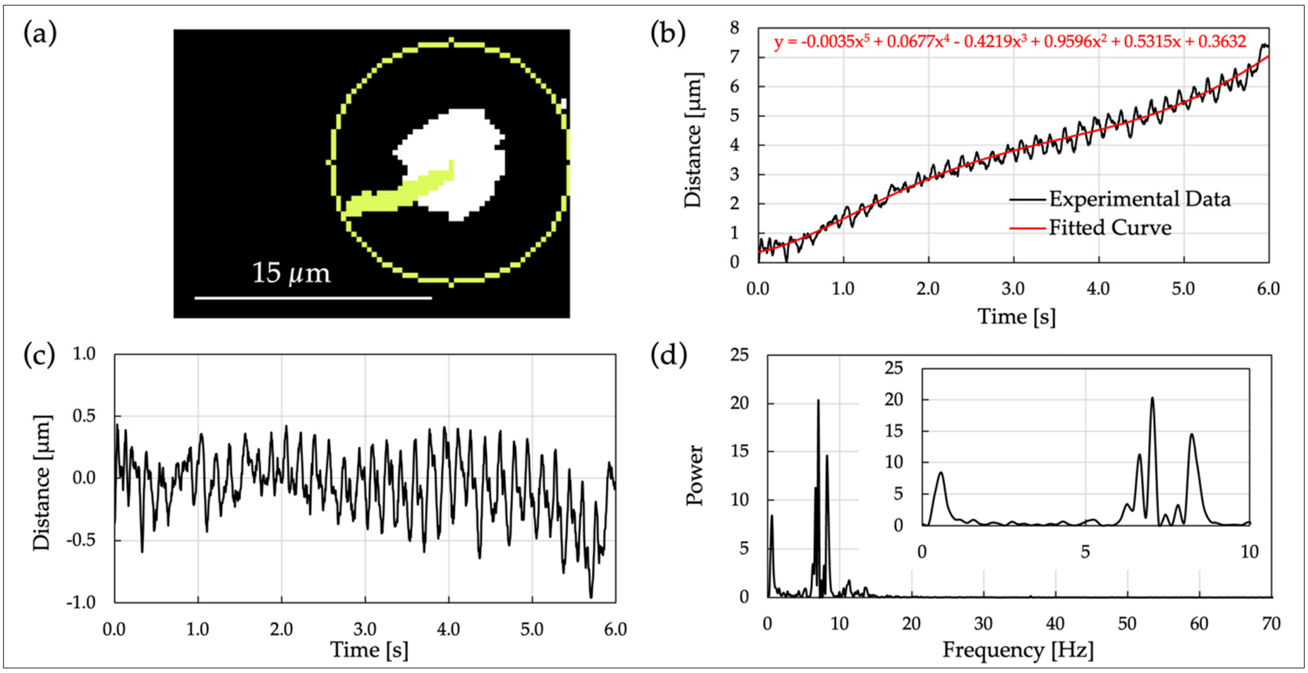

Figure S6. Analysis of cell motility at low temperature (0.4 °C) using a green LED as a light source (Figure 5f): (a) Trajectory of cell motility; (b) Change in cell swimming distance over time; (c) Difference between the measured value and the approximate curve in (b); (d) FFT analysis result of (c)

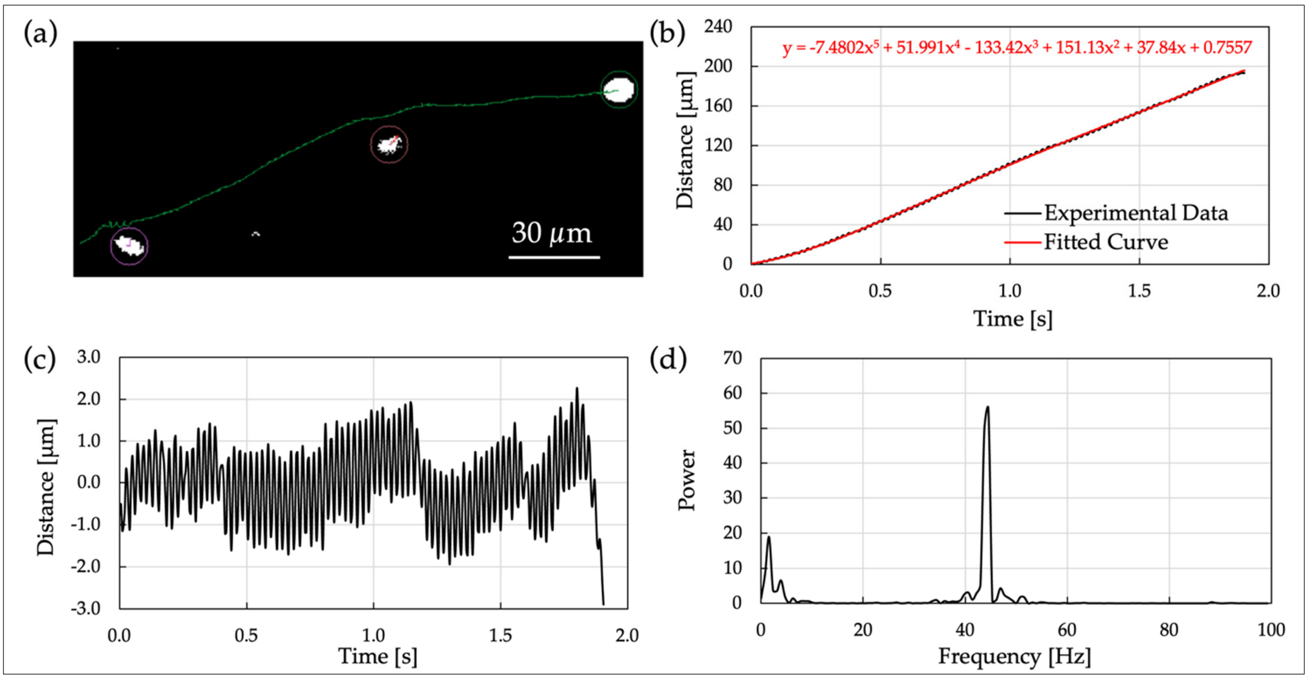

Figure S7. Analysis of cell motility at room temperature (21.4 °C) using a blue LED as a light source (Figure 5g): (a) Trajectory of cell motility; (b) Change in cell swimming distance over time; (c) Difference between the measured value and the approximate curve in (b); (d) FFT analysis result of (c)

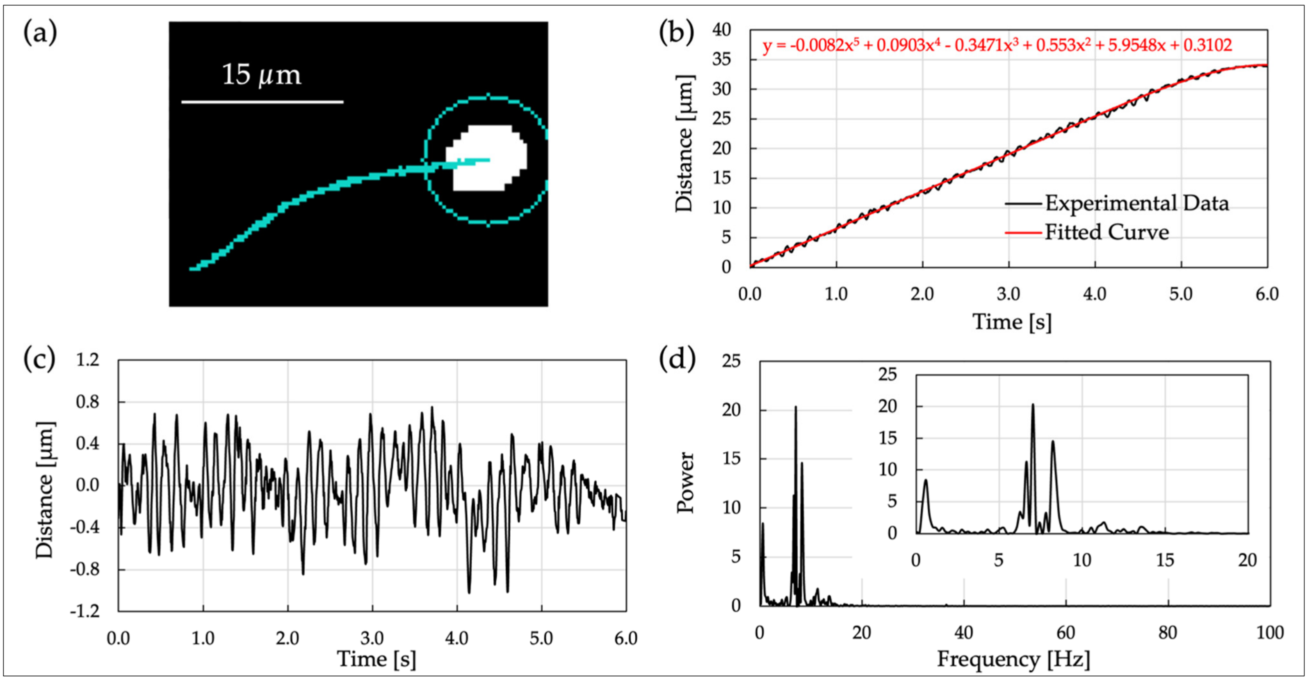

Figure S8. Analysis of cell motility at low temperature (1.7 °C) using a blue LED as a light source (Figure 5h): (a) Trajectory of cell motility; (b) Change in cell swimming distance over time; (c) Difference between the measured value and the approximate curve in (b); (d) FFT analysis result of (c)

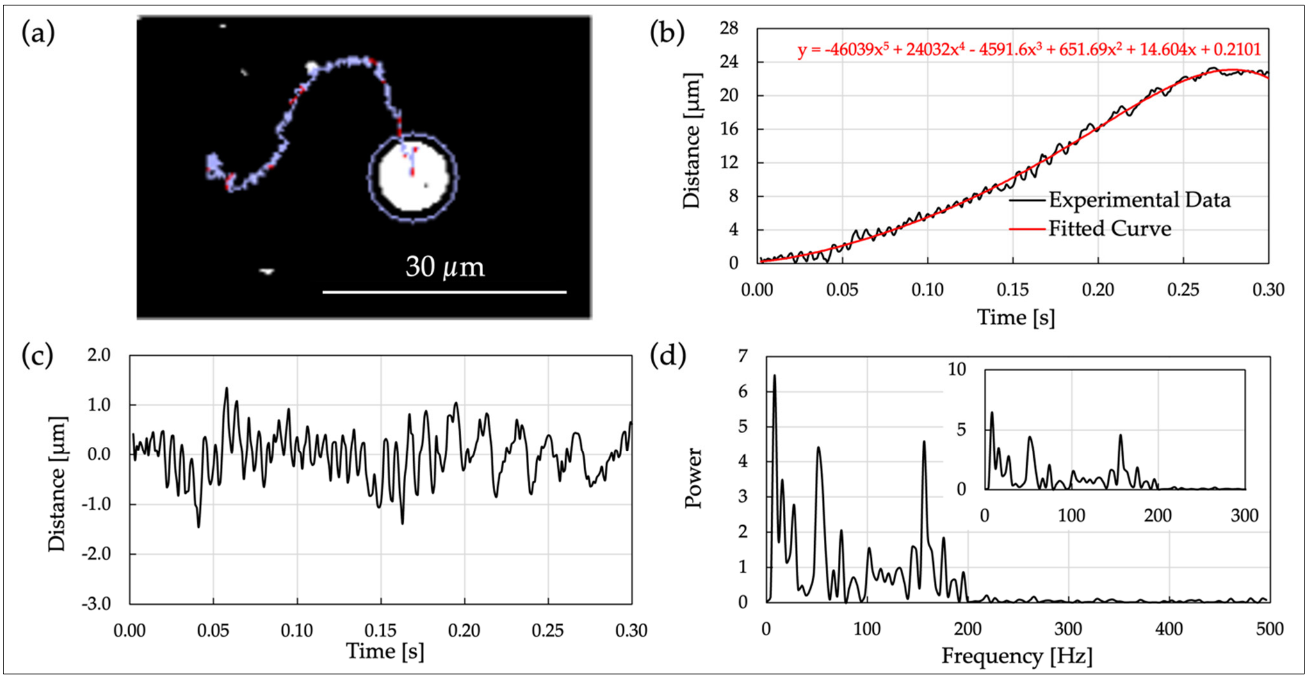

Figure S9. Analysis of cell motility in viscous solution (Ficoll 7%, 3.5 mPa·s) at room temperature (24.0 °C) using a halogen lamp as a light source (Figure 6a): (a) Trajectory of cell motility; (b) Change in cell swimming distance over time; (c) Difference between the measured value and the approximate curve in (b); (d) FFT analysis result of (c)

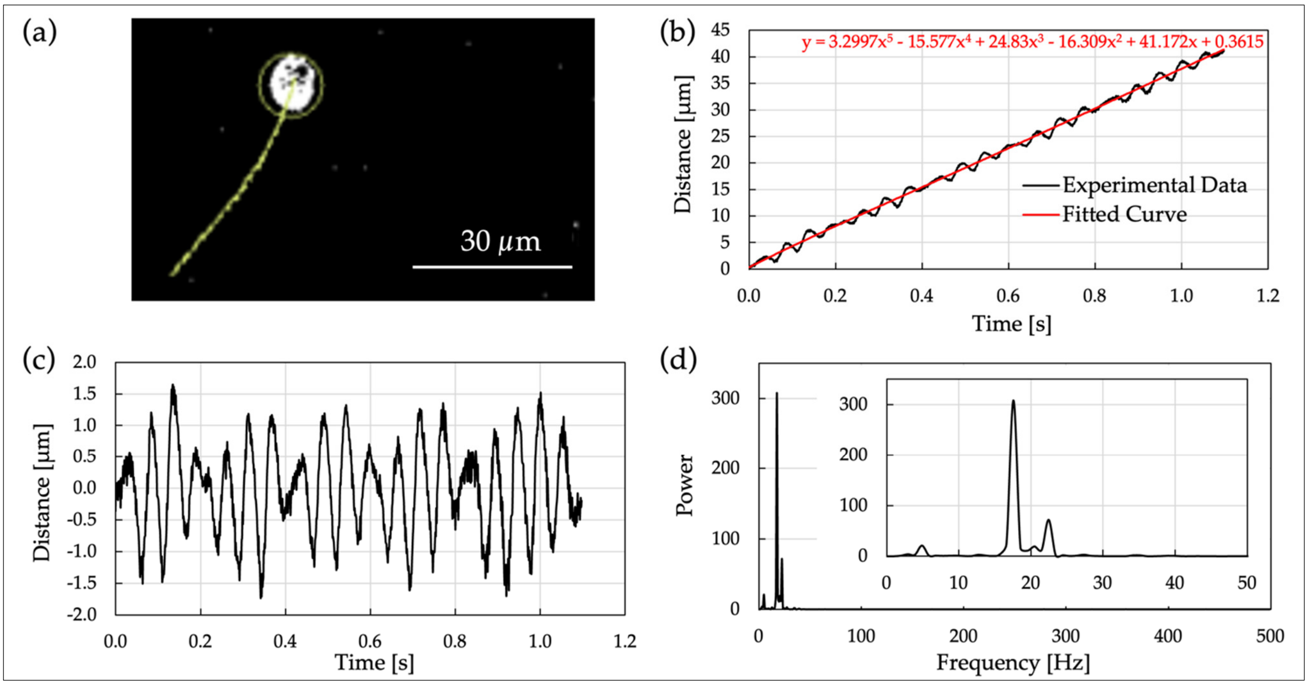

Figure S10. Analysis of cell motility at low temperature (−4 °C) using a halogen lamp as a light source (Figure 6b): (a) Trajectory of cell motility; (b) Change in cell swimming distance over time; (c) Difference between the measured value and the approximate curve in (b); (d) FFT analysis result of (c)

2. The temporal change in average velocity histogram of cells over time

Histograms of the average velocity of cells at each elapsed time in Figure 4a of the main text were created. The results are shown in Figure S11.

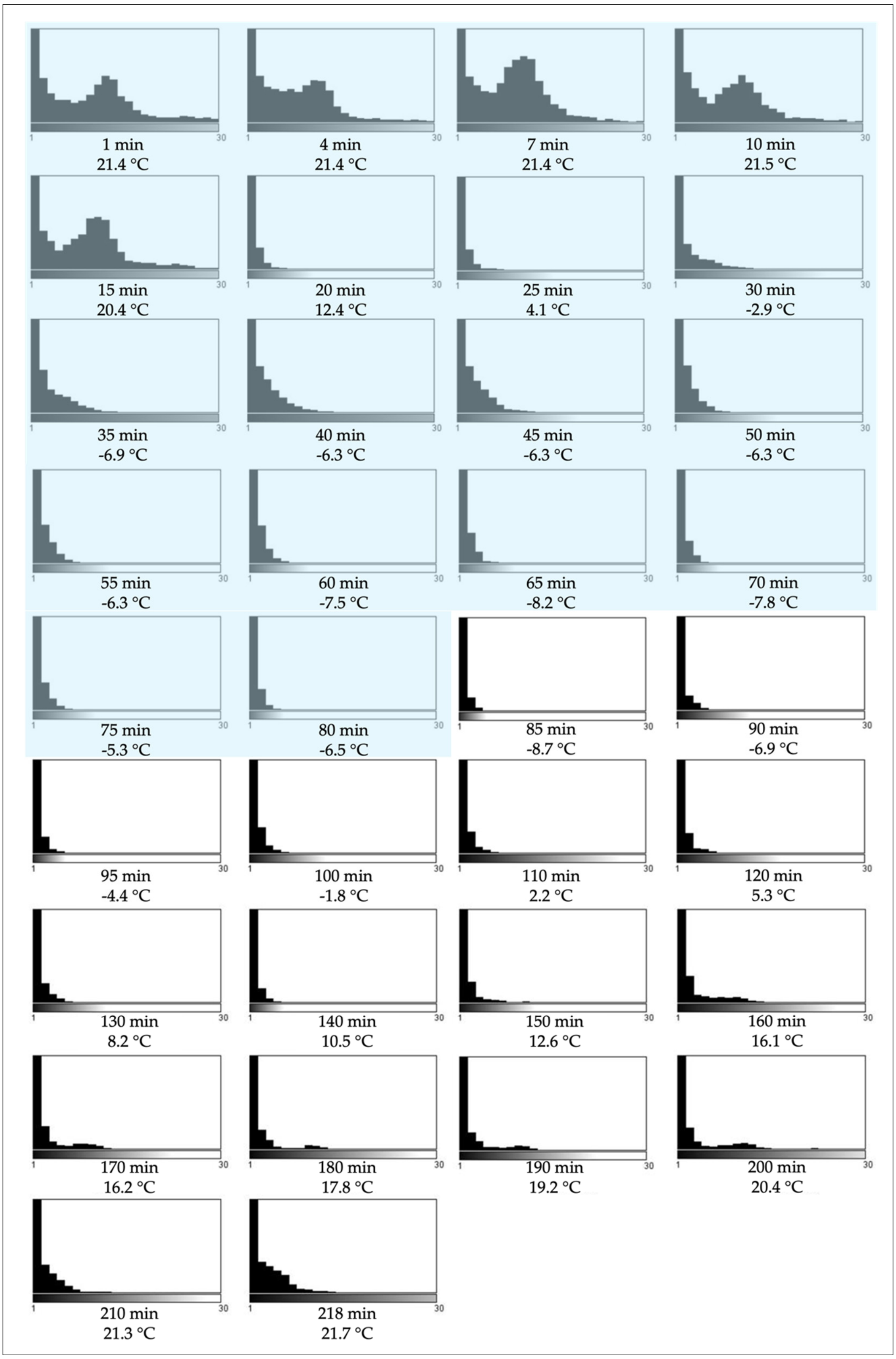

Figure S11. Histogram of average velocity of cells (Figure 4a): In each histogram, the horizontal axis shows the average velocity at the center of cell (μm/s), and the vertical axis shows the frequency. The time (min) written directly below each

histogram indicates the elapsed time from the start of measurement. The light blue highlighted time period is the same time period as the light blue highlighted part in Figure 4a (the period from the start until the temperature decreases and rises again).
